# Support for current patients infected addicts

**DOI:** 10.1186/1471-2334-14-S2-P55

**Published:** 2014-05-23

**Authors:** Karine Bartolo, Pascal Auquier

**Affiliations:** 1 Aix-Marseille University, La Timone Faculty, Addiction Center, Marseille, France

## 

In 2013, the network supported 282 addict patients. 121 patients including 24 women are infected with HIV or HCV and 109 have hepatitis C. All have a substitution of Buprenorphine or Methadone treatment. 110 / 121 patients have a general practitioner and 101 / 121 have a regular pharmacist. (figure1)

**Figure 1:**
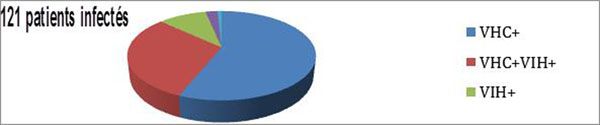


25 patients are cured of hepatitis C out of 47 who received treatment and 14 patients are cured spontaneously without treatment. Among 121 patients, 16 have already been hospitalized in psychiatry. 45 patients have a psychiatric co-morbidity and 40 are treated for this disease. (figure 2)

**Figure 2:**
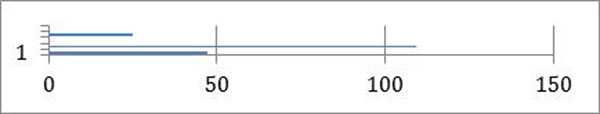


Very few patients have less than 200 CD4. (figure 3)

**Figure 3:**
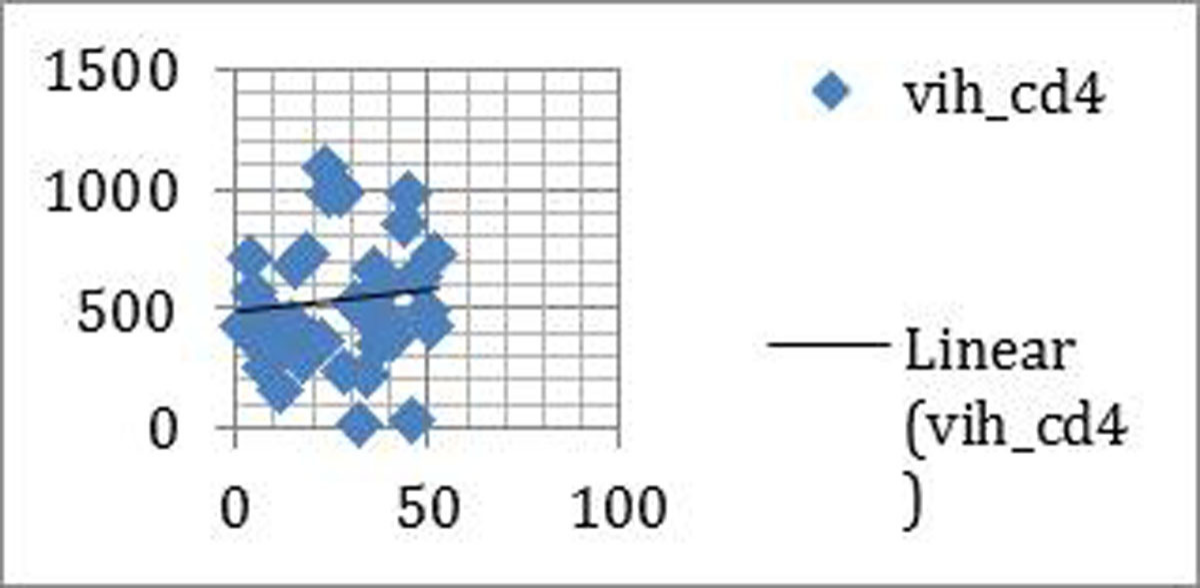


Most patients were followed up since 2008. (figure 4)

**Figure 4:**
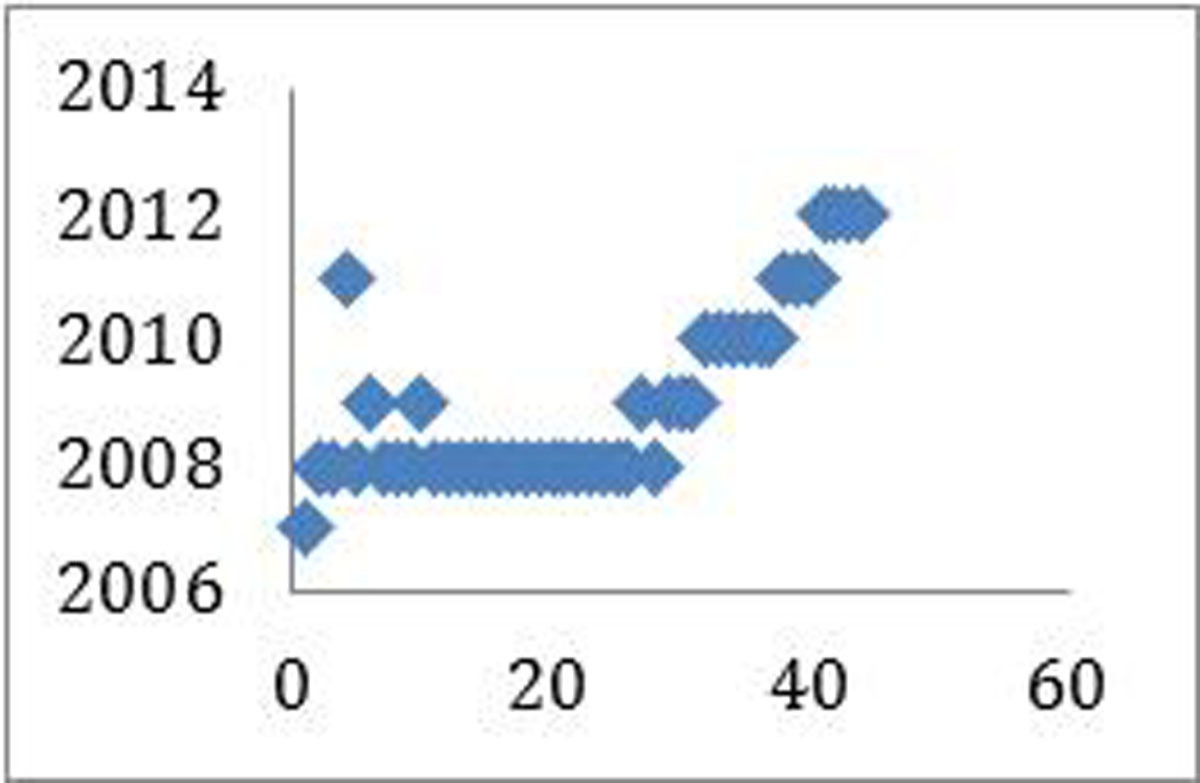


## Conclusion

Addict patients take subscribe to a course of complex care, and often long term. They are often supported in a complementary manner by the city and the hospital. Their immune status for seropositive patients of HIV are often above 200 CD4. They often have an associated psychiatric comorbidity for which they receive treatment. When the treatment with Interferon is go on, only ½ is cured. Today, they most often have non-treated hepatitis C.

